# Supplementary material for: Modeling glioblastoma heterogeneity as a dynamic network of cell states
Source: Mol Syst Biol. 2021 Sep 16;17(9):e10105. doi: 10.15252/msb.202010105 (PMC8444284; doi:10.15252/msb.202010105)
Supplement: Supplementary file 6 — Source Data for Figure 5 [file MSB-17-e10105-s004.zip › Figure5A_sourcedata/GSEA_3071/nosigdb_stateIII.GseaPreranked.1622795155562/RICHARDS_NOWAKOWSKI_SCIENCE_2017_IPC_DIV2_UPREG.html]

Details for gene set RICHARDS\_NOWAKOWSKI\_SCIENCE\_2017\_IPC\_DIV2\_UPREG[GSEA]

|  || Dataset | state33071 |
| Phenotype | NoPhenotypeAvailable |
| Upregulated in class | na\_pos |
| GeneSet | RICHARDS\_NOWAKOWSKI\_SCIENCE\_2017\_IPC\_DIV2\_UPREG |
| Enrichment Score (ES) | 0.137417 |
| Normalized Enrichment Score (NES) | NaN |
| Nominal p-value | NaN |
| FDR q-value | 1.0 |
| FWER p-Value | 0.0 |
Table: GSEA Results Summary

  

Fig 1: Enrichment plot: RICHARDS\_NOWAKOWSKI\_SCIENCE\_2017\_IPC\_DIV2\_UPREG      
 Profile of the Running ES Score & Positions of GeneSet Members on the Rank Ordered List

  

| PROBE | GENE SYMBOL | GENE\_TITLE | RANK IN GENE LIST | RANK METRIC SCORE | RUNNING ES | CORE ENRICHMENT || 1 | RPS26 |  |  | 9 | 0.595 | 0.0027 | Yes |
| 2 | UQCRQ |  |  | 13 | 0.574 | 0.0068 | Yes |
| 3 | ROMO1 |  |  | 14 | 0.569 | 0.0117 | Yes |
| 4 | RPLP1 |  |  | 25 | 0.514 | 0.0133 | Yes |
| 5 | EEF1B2 |  |  | 28 | 0.501 | 0.0171 | Yes |
| 6 | EIF2S2 |  |  | 30 | 0.499 | 0.0211 | Yes |
| 7 | POLR2L |  |  | 38 | 0.482 | 0.0234 | Yes |
| 8 | PFN1 |  |  | 40 | 0.481 | 0.0272 | Yes |
| 9 | RPL8 |  |  | 44 | 0.469 | 0.0304 | Yes |
| 10 | SNRPC |  |  | 46 | 0.466 | 0.0342 | Yes |
| 11 | NDUFB2 |  |  | 52 | 0.460 | 0.0368 | Yes |
| 12 | NDUFA6 |  |  | 54 | 0.458 | 0.0404 | Yes |
| 13 | CYCS |  |  | 59 | 0.453 | 0.0432 | Yes |
| 14 | COX8A |  |  | 63 | 0.448 | 0.0463 | Yes |
| 15 | RPS24 |  |  | 69 | 0.444 | 0.0487 | Yes |
| 16 | SLIRP |  |  | 80 | 0.430 | 0.0497 | Yes |
| 17 | MRPL33 |  |  | 87 | 0.423 | 0.0516 | Yes |
| 18 | RPS3 |  |  | 90 | 0.420 | 0.0547 | Yes |
| 19 | TOMM5 |  |  | 103 | 0.409 | 0.0549 | Yes |
| 20 | FAU |  |  | 113 | 0.400 | 0.0559 | Yes |
| 21 | PPIA |  |  | 121 | 0.395 | 0.0574 | Yes |
| 22 | SNRPB |  |  | 122 | 0.395 | 0.0608 | Yes |
| 23 | GNG5 |  |  | 139 | 0.382 | 0.0597 | Yes |
| 24 | SMS |  |  | 149 | 0.375 | 0.0604 | Yes |
| 25 | SSBP1 |  |  | 156 | 0.370 | 0.0620 | Yes |
| 26 | SNRPF |  |  | 160 | 0.367 | 0.0643 | Yes |
| 27 | GLO1 |  |  | 161 | 0.367 | 0.0675 | Yes |
| 28 | PSMC3 |  |  | 162 | 0.366 | 0.0706 | Yes |
| 29 | SET |  |  | 163 | 0.366 | 0.0738 | Yes |
| 30 | YBX1 |  |  | 167 | 0.364 | 0.0761 | Yes |
| 31 | PSMB3 |  |  | 171 | 0.361 | 0.0783 | Yes |
| 32 | SNRPG |  |  | 186 | 0.349 | 0.0775 | Yes |
| 33 | CHCHD2 |  |  | 195 | 0.341 | 0.0782 | Yes |
| 34 | RPS14 |  |  | 196 | 0.340 | 0.0812 | Yes |
| 35 | C19orf53 |  |  | 197 | 0.340 | 0.0841 | Yes |
| 36 | EIF5B |  |  | 204 | 0.336 | 0.0853 | Yes |
| 37 | HINT1 |  |  | 205 | 0.335 | 0.0882 | Yes |
| 38 | SUMO3 |  |  | 210 | 0.333 | 0.0900 | Yes |
| 39 | GSTP1 |  |  | 211 | 0.333 | 0.0928 | Yes |
| 40 | POLR2J |  |  | 215 | 0.331 | 0.0949 | Yes |
| 41 | NCL |  |  | 219 | 0.328 | 0.0969 | Yes |
| 42 | PRDX1 |  |  | 220 | 0.328 | 0.0997 | Yes |
| 43 | RANBP1 |  |  | 221 | 0.328 | 0.1025 | Yes |
| 44 | PTMA |  |  | 222 | 0.327 | 0.1053 | Yes |
| 45 | ENO1 |  |  | 241 | 0.314 | 0.1031 | Yes |
| 46 | ACP1 |  |  | 249 | 0.310 | 0.1038 | Yes |
| 47 | LSM4 |  |  | 268 | 0.303 | 0.1015 | Yes |
| 48 | TMSB4X |  |  | 273 | 0.300 | 0.1030 | Yes |
| 49 | ANAPC11 |  |  | 286 | 0.295 | 0.1022 | Yes |
| 50 | RAB8A |  |  | 287 | 0.295 | 0.1047 | Yes |
| 51 | TMEM258 |  |  | 295 | 0.292 | 0.1053 | Yes |
| 52 | TIMM10 |  |  | 304 | 0.290 | 0.1056 | Yes |
| 53 | CMSS1 |  |  | 307 | 0.289 | 0.1076 | Yes |
| 54 | PA2G4 |  |  | 308 | 0.289 | 0.1101 | Yes |
| 55 | POLR2I |  |  | 311 | 0.289 | 0.1120 | Yes |
| 56 | EIF4EBP1 |  |  | 316 | 0.287 | 0.1134 | Yes |
| 57 | BANF1 |  |  | 317 | 0.287 | 0.1158 | Yes |
| 58 | HMGB1 |  |  | 318 | 0.286 | 0.1183 | Yes |
| 59 | GAPDH |  |  | 320 | 0.286 | 0.1205 | Yes |
| 60 | CSNK2B |  |  | 333 | 0.283 | 0.1196 | Yes |
| 61 | SKA2 |  |  | 343 | 0.280 | 0.1196 | Yes |
| 62 | GUK1 |  |  | 349 | 0.276 | 0.1206 | Yes |
| 63 | NAA38 |  |  | 354 | 0.274 | 0.1218 | Yes |
| 64 | MYL6 |  |  | 360 | 0.272 | 0.1228 | Yes |
| 65 | RBM17 |  |  | 364 | 0.271 | 0.1243 | Yes |
| 66 | COX17 |  |  | 368 | 0.270 | 0.1258 | Yes |
| 67 | CLIC1 |  |  | 373 | 0.269 | 0.1270 | Yes |
| 68 | SNRPD1 |  |  | 376 | 0.268 | 0.1288 | Yes |
| 69 | MRPL51 |  |  | 380 | 0.267 | 0.1303 | Yes |
| 70 | H2AFZ |  |  | 386 | 0.265 | 0.1312 | Yes |
| 71 | RAN |  |  | 388 | 0.264 | 0.1332 | Yes |
| 72 | EIF4H |  |  | 389 | 0.264 | 0.1354 | Yes |
| 73 | FAM136A |  |  | 391 | 0.263 | 0.1374 | Yes |
| 74 | ALDOA |  |  | 440 | -0.251 | 0.1264 | No |
| 75 | MNS1 |  |  | 463 | -0.252 | 0.1225 | No |
| 76 | RAD54L |  |  | 497 | -0.254 | 0.1156 | No |
| 77 | MTBP |  |  | 498 | -0.254 | 0.1178 | No |
| 78 | CDC45 |  |  | 506 | -0.255 | 0.1180 | No |
| 79 | HJURP |  |  | 513 | -0.255 | 0.1186 | No |
| 80 | POLE2 |  |  | 520 | -0.255 | 0.1191 | No |
| 81 | INCENP |  |  | 534 | -0.256 | 0.1178 | No |
| 82 | PHIP |  |  | 560 | -0.257 | 0.1131 | No |
| 83 | ARHGAP11B |  |  | 585 | -0.259 | 0.1087 | No |
| 84 | PHLDA1 |  |  | 589 | -0.259 | 0.1101 | No |
| 85 | ZNF43 |  |  | 594 | -0.259 | 0.1113 | No |
| 86 | ZNF726 |  |  | 621 | -0.261 | 0.1064 | No |
| 87 | NDC80 |  |  | 636 | -0.261 | 0.1048 | No |
| 88 | MCM4 |  |  | 638 | -0.261 | 0.1067 | No |
| 89 | ELAVL2 |  |  | 695 | -0.264 | 0.0936 | No |
| 90 | LDLRAD3 |  |  | 698 | -0.264 | 0.0953 | No |
| 91 | PRDX2 |  |  | 708 | -0.265 | 0.0951 | No |
| 92 | SHROOM3 |  |  | 720 | -0.266 | 0.0944 | No |
| 93 | IGF2BP3 |  |  | 754 | -0.267 | 0.0876 | No |
| 94 | POM121C |  |  | 766 | -0.268 | 0.0869 | No |
| 95 | LZTS1 |  |  | 837 | -0.271 | 0.0700 | No |
| 96 | HES6 |  |  | 860 | -0.272 | 0.0662 | No |
| 97 | FUCA2 |  |  | 884 | -0.274 | 0.0623 | No |
| 98 | GNB4 |  |  | 885 | -0.274 | 0.0646 | No |
| 99 | HSPA14 |  |  | 896 | -0.274 | 0.0642 | No |
| 100 | ENOSF1 |  |  | 899 | -0.274 | 0.0660 | No |
| 101 | ADCY3 |  |  | 932 | -0.276 | 0.0596 | No |
| 102 | ATP1B3 |  |  | 937 | -0.276 | 0.0609 | No |
| 103 | C21orf58 |  |  | 946 | -0.276 | 0.0611 | No |
| 104 | DHFR |  |  | 956 | -0.277 | 0.0610 | No |
| 105 | ASPH |  |  | 965 | -0.278 | 0.0612 | No |
| 106 | HELLS |  |  | 970 | -0.278 | 0.0625 | No |
| 107 | C5orf22 |  |  | 1000 | -0.279 | 0.0569 | No |
| 108 | MCM3 |  |  | 1021 | -0.280 | 0.0538 | No |
| 109 | ZNF142 |  |  | 1022 | -0.280 | 0.0562 | No |
| 110 | CDCA3 |  |  | 1029 | -0.281 | 0.0570 | No |
| 111 | SP2 |  |  | 1079 | -0.284 | 0.0459 | No |
| 112 | GPC6 |  |  | 1101 | -0.285 | 0.0426 | No |
| 113 | FOXM1 |  |  | 1102 | -0.285 | 0.0451 | No |
| 114 | RNASEH2A |  |  | 1103 | -0.285 | 0.0475 | No |
| 115 | PCNA |  |  | 1116 | -0.286 | 0.0467 | No |
| 116 | NR2C2 |  |  | 1129 | -0.286 | 0.0458 | No |
| 117 | SPIN4 |  |  | 1179 | -0.289 | 0.0348 | No |
| 118 | SLC39A11 |  |  | 1182 | -0.289 | 0.0368 | No |
| 119 | SUGP2 |  |  | 1194 | -0.290 | 0.0362 | No |
| 120 | RIF1 |  |  | 1214 | -0.291 | 0.0335 | No |
| 121 | NUSAP1 |  |  | 1262 | -0.293 | 0.0231 | No |
| 122 | DTL |  |  | 1297 | -0.295 | 0.0163 | No |
| 123 | SLC4A7 |  |  | 1314 | -0.295 | 0.0144 | No |
| 124 | ARHGAP33 |  |  | 1323 | -0.296 | 0.0148 | No |
| 125 | CCDC88C |  |  | 1378 | -0.299 | 0.0025 | No |
| 126 | LAPTM4A |  |  | 1391 | -0.299 | 0.0018 | No |
| 127 | CDON |  |  | 1423 | -0.301 | -0.0042 | No |
| 128 | IGF2R |  |  | 1432 | -0.302 | -0.0038 | No |
| 129 | XRCC2 |  |  | 1435 | -0.302 | -0.0017 | No |
| 130 | GGH |  |  | 1439 | -0.302 | 0.0001 | No |
| 131 | CREB5 |  |  | 1470 | -0.304 | -0.0056 | No |
| 132 | CIPC |  |  | 1478 | -0.305 | -0.0049 | No |
| 133 | MMS22L |  |  | 1494 | -0.306 | -0.0064 | No |
| 134 | KIF4A |  |  | 1504 | -0.306 | -0.0062 | No |
| 135 | TGIF1 |  |  | 1506 | -0.306 | -0.0038 | No |
| 136 | SEC63 |  |  | 1534 | -0.308 | -0.0086 | No |
| 137 | EXO1 |  |  | 1537 | -0.308 | -0.0065 | No |
| 138 | LINC01224 |  |  | 1538 | -0.308 | -0.0039 | No |
| 139 | DBR1 |  |  | 1543 | -0.308 | -0.0023 | No |
| 140 | ZEB1 |  |  | 1569 | -0.310 | -0.0065 | No |
| 141 | MYBL2 |  |  | 1587 | -0.311 | -0.0085 | No |
| 142 | MED12L |  |  | 1593 | -0.311 | -0.0072 | No |
| 143 | DAG1 |  |  | 1598 | -0.312 | -0.0056 | No |
| 144 | GTF2E1 |  |  | 1629 | -0.314 | -0.0112 | No |
| 145 | PLK4 |  |  | 1642 | -0.315 | -0.0118 | No |
| 146 | ZC3H12C |  |  | 1673 | -0.317 | -0.0173 | No |
| 147 | C3orf70 |  |  | 1698 | -0.318 | -0.0212 | No |
| 148 | GNG4 |  |  | 1706 | -0.319 | -0.0203 | No |
| 149 | ZNF714 |  |  | 1709 | -0.319 | -0.0181 | No |
| 150 | CHEK2 |  |  | 1718 | -0.320 | -0.0176 | No |
| 151 | HAUS8 |  |  | 1741 | -0.321 | -0.0209 | No |
| 152 | NUF2 |  |  | 1755 | -0.322 | -0.0217 | No |
| 153 | CUX1 |  |  | 1776 | -0.323 | -0.0244 | No |
| 154 | WDR76 |  |  | 1795 | -0.324 | -0.0266 | No |
| 155 | PLK1 |  |  | 1828 | -0.326 | -0.0326 | No |
| 156 | GLI2 |  |  | 1843 | -0.327 | -0.0336 | No |
| 157 | BRCA1 |  |  | 1844 | -0.327 | -0.0308 | No |
| 158 | HIP1 |  |  | 1905 | -0.330 | -0.0445 | No |
| 159 | DAAM1 |  |  | 1915 | -0.330 | -0.0441 | No |
| 160 | STARD9 |  |  | 1918 | -0.330 | -0.0418 | No |
| 161 | EFCAB2 |  |  | 1922 | -0.331 | -0.0398 | No |
| 162 | UBE2C |  |  | 1942 | -0.332 | -0.0421 | No |
| 163 | SALL1 |  |  | 1962 | -0.333 | -0.0445 | No |
| 164 | SMC2 |  |  | 1963 | -0.333 | -0.0416 | No |
| 165 | LBR |  |  | 1973 | -0.334 | -0.0412 | No |
| 166 | PKD2 |  |  | 1977 | -0.334 | -0.0392 | No |
| 167 | SLC16A1 |  |  | 2012 | -0.336 | -0.0456 | No |
| 168 | MYO1E |  |  | 2017 | -0.337 | -0.0438 | No |
| 169 | DNA2 |  |  | 2031 | -0.337 | -0.0445 | No |
| 170 | NLGN1 |  |  | 2107 | -0.342 | -0.0622 | No |
| 171 | CENPI |  |  | 2121 | -0.343 | -0.0628 | No |
| 172 | RRM2 |  |  | 2147 | -0.345 | -0.0667 | No |
| 173 | MFAP2 |  |  | 2169 | -0.347 | -0.0695 | No |
| 174 | LIN9 |  |  | 2212 | -0.350 | -0.0781 | No |
| 175 | BRCA2 |  |  | 2213 | -0.350 | -0.0751 | No |
| 176 | ZNF90 |  |  | 2220 | -0.350 | -0.0737 | No |
| 177 | RNF165 |  |  | 2247 | -0.352 | -0.0778 | No |
| 178 | FAM149B1 |  |  | 2287 | -0.356 | -0.0855 | No |
| 179 | TPX2 |  |  | 2289 | -0.356 | -0.0827 | No |
| 180 | KIF26B |  |  | 2291 | -0.356 | -0.0799 | No |
| 181 | ZNF367 |  |  | 2292 | -0.356 | -0.0768 | No |
| 182 | MCM2 |  |  | 2303 | -0.357 | -0.0765 | No |
| 183 | FRMD5 |  |  | 2309 | -0.358 | -0.0748 | No |
| 184 | ASF1B |  |  | 2312 | -0.358 | -0.0723 | No |
| 185 | BSG |  |  | 2316 | -0.359 | -0.0700 | No |
| 186 | MAGI1 |  |  | 2340 | -0.361 | -0.0732 | No |
| 187 | DEPDC1B |  |  | 2354 | -0.362 | -0.0737 | No |
| 188 | ENO2 |  |  | 2366 | -0.363 | -0.0736 | No |
| 189 | SPECC1 |  |  | 2368 | -0.363 | -0.0707 | No |
| 190 | POLQ |  |  | 2401 | -0.365 | -0.0764 | No |
| 191 | IQGAP1 |  |  | 2405 | -0.366 | -0.0741 | No |
| 192 | DLGAP5 |  |  | 2421 | -0.367 | -0.0750 | No |
| 193 | PSD3 |  |  | 2464 | -0.371 | -0.0834 | No |
| 194 | DENND5B |  |  | 2496 | -0.373 | -0.0887 | No |
| 195 | GTF2IRD1 |  |  | 2501 | -0.373 | -0.0866 | No |
| 196 | ZXDC |  |  | 2532 | -0.376 | -0.0916 | No |
| 197 | SDC2 |  |  | 2537 | -0.376 | -0.0895 | No |
| 198 | CDC7 |  |  | 2539 | -0.376 | -0.0865 | No |
| 199 | TACC3 |  |  | 2560 | -0.379 | -0.0888 | No |
| 200 | KIF20A |  |  | 2561 | -0.379 | -0.0855 | No |
| 201 | BAZ2B |  |  | 2576 | -0.380 | -0.0861 | No |
| 202 | TMTC4 |  |  | 2583 | -0.380 | -0.0845 | No |
| 203 | FAM111B |  |  | 2585 | -0.381 | -0.0815 | No |
| 204 | FADS1 |  |  | 2588 | -0.381 | -0.0787 | No |
| 205 | IQGAP3 |  |  | 2592 | -0.381 | -0.0763 | No |
| 206 | TMTC2 |  |  | 2616 | -0.383 | -0.0793 | No |
| 207 | SPIDR |  |  | 2618 | -0.383 | -0.0763 | No |
| 208 | PAX6 |  |  | 2619 | -0.384 | -0.0730 | No |
| 209 | CCNA2 |  |  | 2635 | -0.385 | -0.0738 | No |
| 210 | MDM1 |  |  | 2643 | -0.385 | -0.0724 | No |
| 211 | KIF20B |  |  | 2689 | -0.390 | -0.0814 | No |
| 212 | CDCA7 |  |  | 2710 | -0.391 | -0.0836 | No |
| 213 | E2F8 |  |  | 2724 | -0.393 | -0.0838 | No |
| 214 | NEK2 |  |  | 2758 | -0.395 | -0.0894 | No |
| 215 | NOTCH3 |  |  | 2759 | -0.395 | -0.0860 | No |
| 216 | FAM111A |  |  | 2761 | -0.396 | -0.0829 | No |
| 217 | KIF18B |  |  | 2867 | -0.405 | -0.1083 | No |
| 218 | SMC4 |  |  | 2872 | -0.405 | -0.1059 | No |
| 219 | ELOVL2 |  |  | 2879 | -0.406 | -0.1041 | No |
| 220 | C2CD3 |  |  | 2886 | -0.407 | -0.1022 | No |
| 221 | ZGRF1 |  |  | 2897 | -0.408 | -0.1015 | No |
| 222 | RBM15 |  |  | 2901 | -0.408 | -0.0988 | No |
| 223 | AASS |  |  | 2929 | -0.412 | -0.1027 | No |
| 224 | RHOBTB3 |  |  | 2955 | -0.415 | -0.1060 | No |
| 225 | CSRP2 |  |  | 2974 | -0.417 | -0.1073 | No |
| 226 | FANCA |  |  | 2976 | -0.417 | -0.1040 | No |
| 227 | ASRGL1 |  |  | 2986 | -0.418 | -0.1029 | No |
| 228 | RTTN |  |  | 2999 | -0.420 | -0.1026 | No |
| 229 | CRLF3 |  |  | 3015 | -0.422 | -0.1031 | No |
| 230 | CCND2 |  |  | 3037 | -0.425 | -0.1052 | No |
| 231 | FOXRED2 |  |  | 3046 | -0.425 | -0.1037 | No |
| 232 | FANCD2 |  |  | 3065 | -0.427 | -0.1050 | No |
| 233 | CCDC150 |  |  | 3069 | -0.428 | -0.1022 | No |
| 234 | TBC1D31 |  |  | 3079 | -0.429 | -0.1009 | No |
| 235 | GPRC5B |  |  | 3092 | -0.430 | -0.1005 | No |
| 236 | TICRR |  |  | 3106 | -0.432 | -0.1004 | No |
| 237 | ATAD2 |  |  | 3115 | -0.433 | -0.0989 | No |
| 238 | MARCH1 |  |  | 3128 | -0.435 | -0.0984 | No |
| 239 | MCM8 |  |  | 3129 | -0.435 | -0.0947 | No |
| 240 | CCNE2 |  |  | 3138 | -0.436 | -0.0931 | No |
| 241 | PLCB1 |  |  | 3189 | -0.441 | -0.1031 | No |
| 242 | MYO10 |  |  | 3196 | -0.442 | -0.1009 | No |
| 243 | NCAPD2 |  |  | 3208 | -0.443 | -0.1001 | No |
| 244 | CDCA2 |  |  | 3212 | -0.444 | -0.0971 | No |
| 245 | ZNF713 |  |  | 3227 | -0.447 | -0.0971 | No |
| 246 | CDCA7L |  |  | 3311 | -0.462 | -0.1160 | No |
| 247 | POLE |  |  | 3335 | -0.468 | -0.1183 | No |
| 248 | ZNF184 |  |  | 3342 | -0.469 | -0.1159 | No |
| 249 | SLC20A1 |  |  | 3344 | -0.470 | -0.1121 | No |
| 250 | CELSR2 |  |  | 3378 | -0.475 | -0.1171 | No |
| 251 | BRIP1 |  |  | 3380 | -0.475 | -0.1133 | No |
| 252 | KIF14 |  |  | 3384 | -0.476 | -0.1100 | No |
| 253 | OTUD3 |  |  | 3430 | -0.487 | -0.1182 | No |
| 254 | EGFR |  |  | 3433 | -0.488 | -0.1146 | No |
| 255 | RNF38 |  |  | 3454 | -0.494 | -0.1158 | No |
| 256 | ZNF606 |  |  | 3456 | -0.494 | -0.1118 | No |
| 257 | GTSE1 |  |  | 3468 | -0.498 | -0.1106 | No |
| 258 | KDM6A |  |  | 3476 | -0.500 | -0.1082 | No |
| 259 | OMD |  |  | 3481 | -0.501 | -0.1050 | No |
| 260 | NCAPG |  |  | 3513 | -0.512 | -0.1091 | No |
| 261 | SYNE2 |  |  | 3523 | -0.518 | -0.1071 | No |
| 262 | F2R |  |  | 3538 | -0.523 | -0.1065 | No |
| 263 | ZFHX4 |  |  | 3540 | -0.524 | -0.1022 | No |
| 264 | CDC25C |  |  | 3551 | -0.526 | -0.1004 | No |
| 265 | CALU |  |  | 3566 | -0.533 | -0.0997 | No |
| 266 | CKB |  |  | 3572 | -0.536 | -0.0965 | No |
| 267 | ITGB1 |  |  | 3574 | -0.537 | -0.0921 | No |
| 268 | RPN2 |  |  | 3581 | -0.539 | -0.0891 | No |
| 269 | CHD7 |  |  | 3622 | -0.561 | -0.0953 | No |
| 270 | ANLN |  |  | 3625 | -0.562 | -0.0910 | No |
| 271 | HIST1H1D |  |  | 3637 | -0.569 | -0.0891 | No |
| 272 | SIPA1L2 |  |  | 3650 | -0.579 | -0.0875 | No |
| 273 | TOP2A |  |  | 3671 | -0.594 | -0.0878 | No |
| 274 | SEL1L3 |  |  | 3686 | -0.604 | -0.0865 | No |
| 275 | CCNF |  |  | 3690 | -0.608 | -0.0821 | No |
| 276 | CA12 |  |  | 3694 | -0.611 | -0.0776 | No |
| 277 | CDK6 |  |  | 3700 | -0.614 | -0.0737 | No |
| 278 | CENPE |  |  | 3711 | -0.625 | -0.0711 | No |
| 279 | AIF1L |  |  | 3725 | -0.635 | -0.0692 | No |
| 280 | MPPED2 |  |  | 3736 | -0.655 | -0.0663 | No |
| 281 | STON2 |  |  | 3739 | -0.658 | -0.0612 | No |
| 282 | FBLN1 |  |  | 3744 | -0.664 | -0.0566 | No |
| 283 | HIST1H1B |  |  | 3749 | -0.669 | -0.0519 | No |
| 284 | MMP16 |  |  | 3753 | -0.675 | -0.0469 | No |
| 285 | POU3F3 |  |  | 3769 | -0.701 | -0.0450 | No |
| 286 | INSM1 |  |  | 3772 | -0.704 | -0.0395 | No |
| 287 | SALL3 |  |  | 3787 | -0.720 | -0.0372 | No |
| 288 | MKI67 |  |  | 3788 | -0.721 | -0.0310 | No |
| 289 | LRIG3 |  |  | 3800 | -0.745 | -0.0276 | No |
| 290 | WSCD1 |  |  | 3835 | -0.798 | -0.0300 | No |
| 291 | NKAIN3 |  |  | 3836 | -0.803 | -0.0231 | No |
| 292 | CENPF |  |  | 3842 | -0.820 | -0.0174 | No |
| 293 | ASCL1 |  |  | 3852 | -0.843 | -0.0127 | No |
| 294 | ASPM |  |  | 3899 | -1.166 | -0.0153 | No |
| 295 | NFIA |  |  | 3910 | -1.317 | -0.0067 | No |
| 296 | HIST1H4C |  |  | 3914 | -1.352 | 0.0041 | No |
Table: GSEA details [plain text format]

  

Fig 2: RICHARDS\_NOWAKOWSKI\_SCIENCE\_2017\_IPC\_DIV2\_UPREG: Random ES distribution      
 Gene set null distribution of ES for **RICHARDS\_NOWAKOWSKI\_SCIENCE\_2017\_IPC\_DIV2\_UPREG**
